# The Medical Treatment of Inebriety

**Published:** 1897-03

**Authors:** 


### THE MEDICAL TREATMENT OF INEBRIETY.

T. D. Crothers, of Hartford, Connecticut, entered a plea for a more careful and general study of inebriety by physicians, particularly as regarded the etiological factors entering into individual cases. He said that the removal of all spirits at the beginning of the treatment was always followed by the best results, and the reaction could be effectually neutralized by giving $\frac{1}{20}$ grain (0.003 gramme) of nitrate of strychnine every four hours, with the bromides, for the relief of insomnia. This treatment should be supplemented by a calomel purge, the use of hot baths, and vigorous massage, and by the administration, at stated intervals, of such nourishment as hot broths. Where a gradual withdrawal of spirits was insisted upon, the form of spirits should be changed to the wines and beers. It would be found that the withdrawal of liquor in these cases often resulted in the sudden and unexpected development of grave organic diseases—*e. g.*, tuberculosis and dementia.

Inebriety was not infrequently the result of an unusual and prolonged strain, or was due to climatic conditions and environment; hence, when such factors are present, every effort should be made to eliminate these factors; otherwise, there would be a speedy relapse. Some of the preparations were useful in averting the "drink-storm," but the administration of drugs, like apomorphine, with the idea of causing a disgust for liquor, was always dangerous, and but rarely satisfactory. The keynote to the successful treatment of these cases was to be found in sharp elimination. Where the bromides were indicated, they should be given in large doses—from 50 to 100 grains(3.25 to 6.50 grammes); these doses should not be continued for more than a few days. No one remedy was capable of meeting a wider range of conditions than the Turkish bath associated with massage.—*Universal Medical Journal.*

